# MaDDFLOSY(Mass Determination via Diffusion in FLow Ordered SpectroscopY) for the Determination of Diffusion-Averaged Molecular Weight of Polymers in Continuous Motion Using Benchtop NMR

**DOI:** 10.1021/acs.macromol.4c03260

**Published:** 2025-04-16

**Authors:** William Pointer, Owen Tooley, Asad Saib, Rowan Radmall, Paul Wilson, Daniel Lester, James Town, Robin J. Blagg, David Haddleton

**Affiliations:** † Department of Chemistry, 2707 University of Warwick, Coventry CV47AL, U.K.; ‡ Oxford Instruments, Halifax Road High Wycombe, Buckinghamshire HP12 3SE, U.K.; § Polymer Characterization RTP, University of Warwick, Coventry CV4 7AL, U.K.

## Abstract

Real-time determination of the molecular weight of polymers synthesized in continuous flow or indeed in any process is essential for efficient and sustainable process chemistry. Typically achieved through online chromatographic techniques, such methods are often prone to perturbations, require large volumes of solvents, have lengthy acquisition times, and can result in significant process inefficiencies. We demonstrate the use of diffusion-ordered NMR spectroscopy (DOSY NMR) calibration on a 60 MHz benchtop to measure the molecular weight of polymers while in laminar flow. We then utilized this technique to monitor batch polymerization progress in real time.

## Graphical abstract

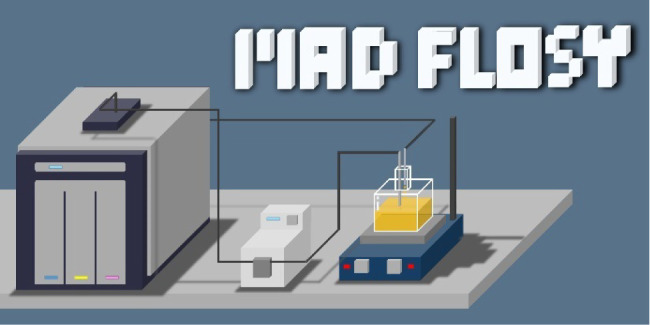

## Introduction

The accurate real-time determination of the molecular mass of polymers, macromolecules, and biomacromolecules is highly beneficial within any manufacturing or research setting involved in high-mass molecules and products. This capability becomes increasingly important with the growing trend toward machine learning and automated approaches for conducting and optimizing research. With this trend, the need for robust analytical techniques becomes an ever more critical factor in producing valuable data sets.^1^

Currently, the most widely used method for molecular weight determination is gel permeation chromatography (GPC). However, online examples of GPC are rare, requiring highly specialized, complex equipment.^2–6^ Additionally, GPC suffers from several key limitations, namely: often poor compatibility with polymers that are difficult to solubilize, relatively long acquisition times (typically 15–60 min) and the requirement for rigorous sample filtration.^3^ Furthermore, as with any chromatographic technique, there exists a complex array of variables that can perturb a measurement, including baseline inconsistencies, solvent contamination, column fouling, interaction, and multiple instrument-dependent factors.

Alternative techniques have been developed for the monitoring of polymerization reactions, particularly the use of inline and online NMR.^7–11^ Both low-field benchtop and high-field instruments have been used to great effect to monitor polymerization progress by tracking the relative integrals of monomers and polymers, providing monomer conversion data.^12,13^ This approach, while insightful for attaining conversion data, struggles to provide adequate data for polymer molecular weight and information about the product molecular characteristics, particularly when end-group fidelity is lost.

Online reaction monitoring is an adaptable quantitative approach that can be used to monitor batch reactions without the need for specialized flow reactors, making it independent of scale. In this method, the reaction mixture is continuously pumped from the reaction vessel through a detector region in the spectrometer, then circulated back to the vessel.^7^ For benchtop NMR, this often involves a tubular cell fitted through the detector region, where a pump maintains the circulation of the reaction mixture.^14,15^

Real-time monitoring is especially effective in reactions carried out under continuous flow conditions, where the product of the reaction is monitored after leaving the reactor.^16^ In this way, real-time data can be used to dynamically optimize processes using machine learning to achieve the targeted parameters.^10^ This is an effective and sustainable method for all chemical processes.

Diffusion-ordered NMR spectroscopy (DOSY) is a valuable technique that provides facile access to an analyte’s diffusion coefficient. This is especially useful to polymer chemists due to the relationship between a polymer’s mass and its rate of diffusion in solution.^17–19^ DOSY NMR conducted on samples in flow would therefore be a desirable evolution of the technique, allowing for online monitoring of polymerization reactions without the need for GPC.^18^ Examples of this have previously been demonstrated using high-field instruments; however, there is no record of this being accomplished on a low-field instrument.^14,20^

Our previous work using a benchtop NMR spectrometer demonstrated the use of a universal mass-determining diffusion-ordered spectroscopy (MaDDOSY) calibration to both accurately determine the molecular weight of a variety of polymers and monitor polymerization reactions in real time.^17,18^ However, a limitation of this work was the necessity to stop the flow during diffusion data acquisition, which limits its application in continuous flow regimes. Herein, we present a further development of the MaDDOSY approach for molecular mass determination and detail methodologies to attain molecular weights of polymers in real time while in continuous motion, without the need to stop the flow of the sample whatsoever.^21^

The approach of measuring molecular weight via DOSY uses the diffusion coefficient, which is related to the hydrodynamic radius of the analyte through the Stokes–Einstein relationship (eq 1), which is, in turn, related to the molecular weight through an adapted Rouse–Zimm model (eq 2).^19,22^

The Stokes-Einstein Relation^23^

1 $$D=\frac{k_{B}T}{6\pi \eta r_{h}}$$

Modified Rouse-Zimm Model.

2 $$r_{h}\approx bM_{D}^{v}$$

The mass obtained in this manner is the diffusion-averaged mass, *M*_D_. This mass should be viewed as a complementary measurement to the values of *M*_n_, *M*_w_, and *M*_v_ obtained through more traditional polymer analysis. Therefore, comparisons made between *M*_D_ and *M*_n_, *M*_w_, and *M*_v_ must be done cautiously, as a separate physical phenomenon is being measured.^24^ It is also noted that *M*_D_ does not provide information about the mass dispersity of the polymer.

While previous work was limited to “stop-flow” conditions or required complex pulse sequences only available on high-field instrumentation, recent advances in benchtop NMR hardware and software now enable more complex experiments under continuous flow conditions.^25–27^ The advantages of this are 3-fold. First, benchtop hardware allows for more complex reactions under demanding conditions, such as those typical of flow processes, to be monitored without specialized setups in proximity to high-field instrumentation. Second, benchtop NMR instrumentation is often less expensive than traditional spectrometers, through not only the initial purchase price but also the lack of requirement for regular liquid cryogen refills. Third, due to an external lock, it is not a requirement to use deuterated solvents, widening the applicability of the technique to reactions that use any common laboratory solvent.

Additionally, in previous work, a pulsed gradient stimulated echo (PGSE) sequence was used to acquire the DOSY spectra. While an excellent technique, PGSE did not provide adequate signal strength for reliable DOSY measurements in steady-state flow regimes. The *J*-compensated pulsed gradient spin echo (J-PGSE) sequence reduces peak distortions caused by ^1^H homonuclear *J*-coupling by refocusing the *J*-evolution during the spin echo to provide enhanced sensitivity, which is ideal for steady-state flow analysis under reaction conditions.

## Results and Discussion

Initial tests of continuous flow DOSY were conducted using a *J*-PGSE pulse sequence.

Samples of poly­(methyl methacrylate) (PMMA), polystyrene (PS/PSTY), and poly­(ethylene glycol) (PEG) were flowed through the Oxford Instruments X-Pulse 60 MHz spectrometer using a Masterflex Ismatec Reglo Miniflex Digital (peristaltic) pump. This device was chosen for providing the flow regime, as it is typical of devices used for continuous flow on the laboratory scale. While other devices, such as syringe or piston pumps, could provide a flow regime with lower pulsation, the peristaltic pump offers a more realistic view of how this analysis is likely to perform for researchers in the laboratory, allowing for recirculation. While investigating the effect of pulsation would be academically interesting, we focused our efforts on providing a valuable example using representative experimental setups.

These polymers were chosen as they represent a broad range of solvent compatibilities, molar masses, and dispersity. The masses as determined by conventional GPC are shown in Table 1. In previous work, we have shown the validity of MaDDOSY as a tool for determining molecular masses up to 200 000 g mol^–1^.^17^ While polymers with masses of that order of magnitude have not been investigated here, they would be expected to perform as previously investigated,

**Table 1:** Molecular Weights of Polymers as Determined by GPC/SEC (THF Eluent against ^a^PMMA and ^b^PSTY Narrow Molecular Weight Standards), and by DOSY NMR Experiments from a 500 and 60 MHz NMR Spectrometer with PS, PMMA, and PEG Dissolved in THF-D8, CDCl3, and D2O, Respectively

	*M*_n_ (GPC) g mol^–1^	*M*_w_ (GPC) g mol^–1^	Dispersity (GPC)	*M*_D_ (500 MHz) g mol^–1^	*M*_D_ (60 MHz) g mol^–1^
^a^PMMA	18000	45000	2.50	22000	37000
^b^PS	10000	16700	1.67	13000	7600
PEG	5100	5500	1.08	5500	5400

As an additional point of comparison, DOSY experiments were also performed on each sample using a 500 MHz NMR spectrometer using a standard “ledbpgp2s” pulse sequence. DOSY experiments using the *J*-PGSE pulse sequence were conducted on the samples while held static within the magnetic field to provide a reference diffusion coefficient. These DOSY experiments were performed using different pulse sequences, which may cause some discrepancy between the values obtained.

As the MaDDOSY calibration is viscosity-corrected and tolerant of any good solvent system, each polymer could have been sampled from any of its respective “good solvents.” The concentration of each sample is critical and has been thoroughly explored in our previous work; in this case, the samples were made up well beneath the C* values determined previously.^17^ Specific details of the polymer/solvent system can be found in the ESI. It is also important to note that nondeuterated solvents can be employed in this experimental setup.

In our original work, we defined a calibration curve relating the diffusion coefficient to molecular weight at 26.5 °C; however, the magnet temperature in the spectrometer used in this current work is held at 40 °C. As the temperature of the system is higher than that of the calibration, the measured diffusion coefficient will also be higher (eq 1).^23^ In our previous work, we described the benefits of using a solvent-independent universal calibration, specifically the ease of obtaining an accurate value without having to create calibrations for each polymer currently known to science. Our philosophy extends to variable temperature measurements; it is impractical to create a set of calibrations comprehensive enough to cover all experiments and spectrometers. Therefore, a simple correction can be performed by calculating the hydrodynamic radius using the Stokes–Einstein equation at the experimental temperature (in this case 40 °C) using the solvent viscosity at that temperature,^28^ and subsequently back-calculating the theoretical diffusion constant at 26.5 °C. This value can then be used to calculate the mass of the polymer using MaDDOSY. To confirm the validity of this approach, a sample of PEG was measured at both 40 °C and 26.5 °C with the same correction applied to the measurement taken at 40 °C. The resulting diffusion coefficients showed an agreement of 99%, the full details can be found in the Supporting Information. While unorthodox, this correction only requires the use of the Stokes–Einstein equation and does not necessitate the use of expensive analytical time or the creation of an additional calibration curve. It must be noted that this correction may be reliable only for relatively small temperature differences, as is the case here. The masses shown in Table 1 have had this correction applied.

PEG shows a strong correlation between the three mass values, indicating that the *M*_D_ determined by the benchtop is a valuable measure for this material. Notably, this PEG sample has a low dispersity. The *M*_D_ determined on the 500 MHz spectrometer shows strong agreement with the 60 MHz, while this is not overly remarkable; it must be remembered that two different pulse sequences have been used, and the strong correlation between these two samples may be due more to the low dispersity of the sample rather than global agreement between the two techniques.

In a vein similar to PEG, the *M*_D_ for PMMA falls between the *M*_n_ and *M*_w_. This is a particularly interesting sample to analyze as there is a wide distribution of molecular species with different diffusion coefficients present in the sample. GPC analysis of PMMA shows a mass distribution from 1 to 300 kg mol^–1^ (see Supporting Information). This value emphasizes that the *M*_D_ should be considered as a complementary mass value rather than as an alternative to the traditionally reported masses. There is some variation in the measurement of *M*_D_ for PMMA from the 500 and the 60 MHz spectrometers, and this is interesting for a number of reasons, primarily because it indicates that the agreement between high-field and low-field NMRs is not guaranteed and seems to deviate more as the dispersity of the polymer increases. Significantly more work is required to explore in detail how the mass, dispersity, and pulse sequence interplay to fully understand the limits and benefits of this approach. It must also be remembered that *M*_D_ is neither *M*_n_ nor *M*_w_ and may not correlate with either.

When determining *M*_D_ (60 MHz) for polystyrene (PS), we investigated the use of a nonbackbone hydrogen signal to determine the diffusion coefficient; in this case, the integral of the whole aromatic region was used. The *M*_D_ value obtained from these peaks was lower than expected, likely due to the increased degrees of motion of these pendant groups. This additional motion affects T1 of the polymer, making the polymer appear to diffuse faster and appear as a smaller polymer. It is useful to understand the limitations of this technique; therefore, PS samples were further tested under continuous flow conditions. For totality, when the diffusion constant was fitted globally, using both the benzyl and backbone peaks, as used for the data acquired at 500 MHz, there is once again near-perfect (Table 1) agreement between GPC and MaDDOSY.

Following this, the samples of each PMMA, PS, and PEG were made up at 50 mg/mL in chloroform, dioxane, and water and were subjected to a range of steady-state laminar flow regimes of 0.1, 0.25, 0.5, 1.0, 1.5, 2.0, and 4.0 mL min^–1^, and the diffusion coefficients were determined by *J*-PGSE experiments. These flow rates have previously been shown to be suitable for diffusion experiments in high-field studies.^20^ A master-flex peristaltic pump was used to flow the samples through the cell. The nature of the peristaltic pump results in some degree of pulsatile flow, leading to a variation in sample flow velocities in the active volume during acquisition. This will introduce additional error into the measurement of the diffusion coefficients. As previously stated, a lower pulsation pump could be used, but a peristaltic pump is more representative of real-world conditions, and the investigation of pump type is outside the scope of this communication.^29^

The resulting diffusion coefficients for each polymer are listed in Figure 1. Among these samples, only PS demonstrates any correlation between the experimentally determined diffusion constants and the flow rate of the analyte, with a slight increase in diffusion at higher flow rates, though this correlation is weak. In contrast, PMMA and PEG show no correlation between the flow rate and the diffusion coefficient. Overall, no clear/consistent correlation between the diffusion coefficient and flow rate is observed when considering all three data sets. The PS, PMMA, and PEG measurements had coefficients of variance of 8.82%, 12.5%, and 6.94%, respectively, well within the accepted 10% error range of a typical, conventional GPC experiment (Figure 1). This further demonstrates a lack of correlation across all three data sets with respect to the flow rate. This test has only been used on a limited range of flow rates and only begins to explore this technique’s limits. The results suggest that for the flow rates explored here, there is, in principle, no reason for flow to be stopped during measurements, giving credence to the use of the technique in continuous flow systems. To investigate the reliability of this approach, a series of tests were conducted whereby the samples were flowed at a consistent flow rate through the spectrometer for several hours, with a series of DOSY measurements being conducted consecutively. The error bars for the PEG samples in Figure 1 show the standard deviation of 10 repeat measurements for each condition used.

**Figure 1:**
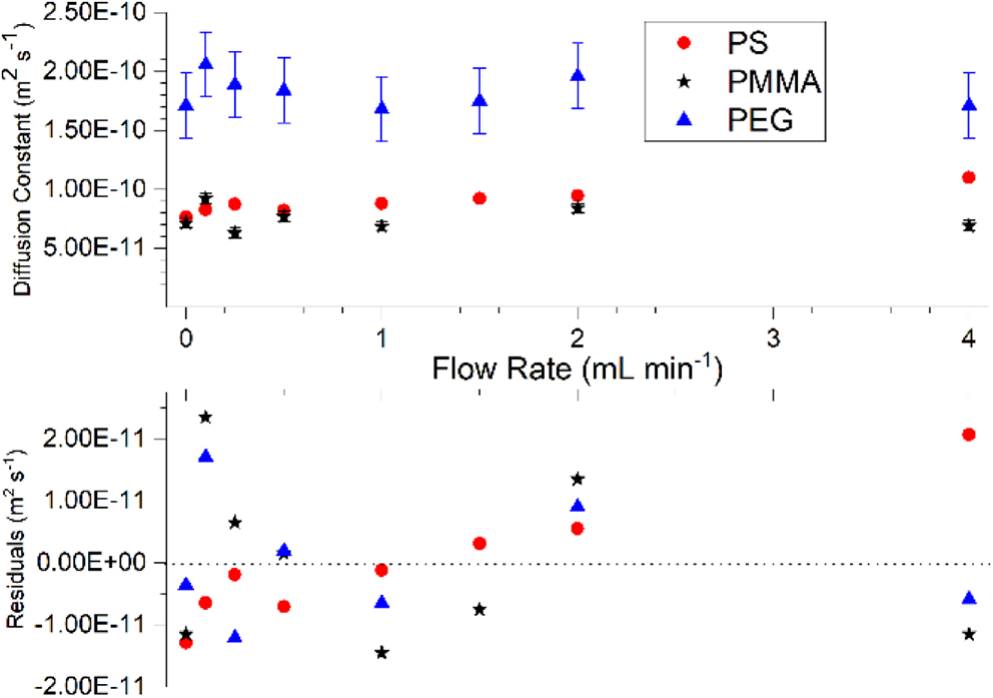
Top- Diffusion coefficients of PMMA (Blue), PEG (Red), and PS (Black) as determined in steady-state continuous flow. Bottom- residuals of each measurement compared to the mean value of each data set.

From these diffusion coefficients, the *M*_D_ value can be determined as previously described (Figure 2). Since there is only a slight variation in the diffusion coefficients across different flow rates, the resulting *M*_D_ values also show minor variation.

**Figure 2:**
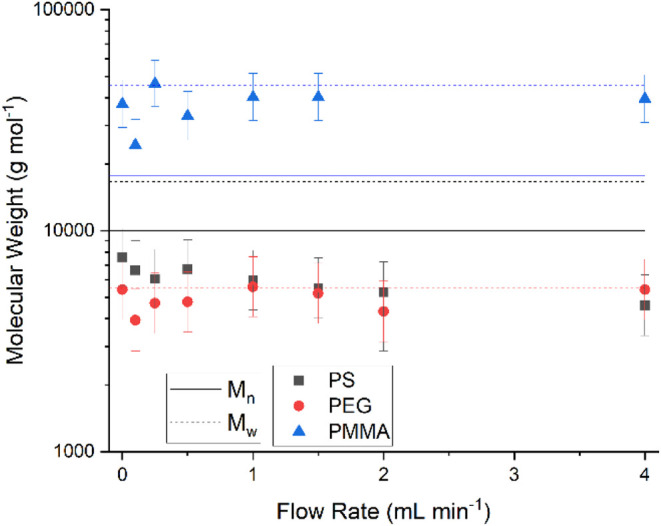
*M*_D_ values for PMMA (blue), PS (black), and PEG (red) as determined at a range of continuous flow rates.

There is again no clear correlation between flow rate and *M*_D_, with the measurements for PS and PEG samples falling within a 10% coefficient of variance of the averaged *M*_D_. For the PS samples, the use of the pendant benzyl groups led to an underestimation of the mass, which was expected following the initial testing. However, this is a strong indicator that nonbackbone hydrogen signals are unsuitable for analysis with this method, as previously discussed. Additionally, the *M*_D_ values for PS show a larger spread compared to the other two samples, though still within a 15% coefficient of variance of the averaged *M*_D_.

To achieve real-time online monitoring with the sample continuously moving through the detector, a well-studied reaction was conducted: RAFT polymerization of methyl acrylate in batch, with the reaction mixture circulating through the system (Figure 3). Full experimental details are available in the Figure S3. Based on the results shown in Figures 1 and 2, a flow rate of 1 mL min^–1^ was selected. Both standard 1D ^1^H and *J*-PGSE DOSY spectra were collected, interleaved with the 1D used for conversion calculations and DOSY for molecular weight calculations. While the temperature used in the thermally initiated RAFT process is higher than the temperature within the spectrometer, it was determined that the reaction mixture was cooled sufficiently by passing through the room-temperature tubing to avoid impacting the measurement. Again, this experimental setup was used to demonstrate how this method can be applied in a practical sense. While the reaction mixture in the vessel was warmer than the spectrometer, the passive cooling through approximately 2 m of tubing was more than sufficient to cool the mixture to room temperature. Additionally, later tests showed that a sample at room temperature passing through the spectrometer at flow rates comparable to ours had a temperature of 40 °C when leaving the spectrometer (see Supporting Information for more details).

**Figure 3:**
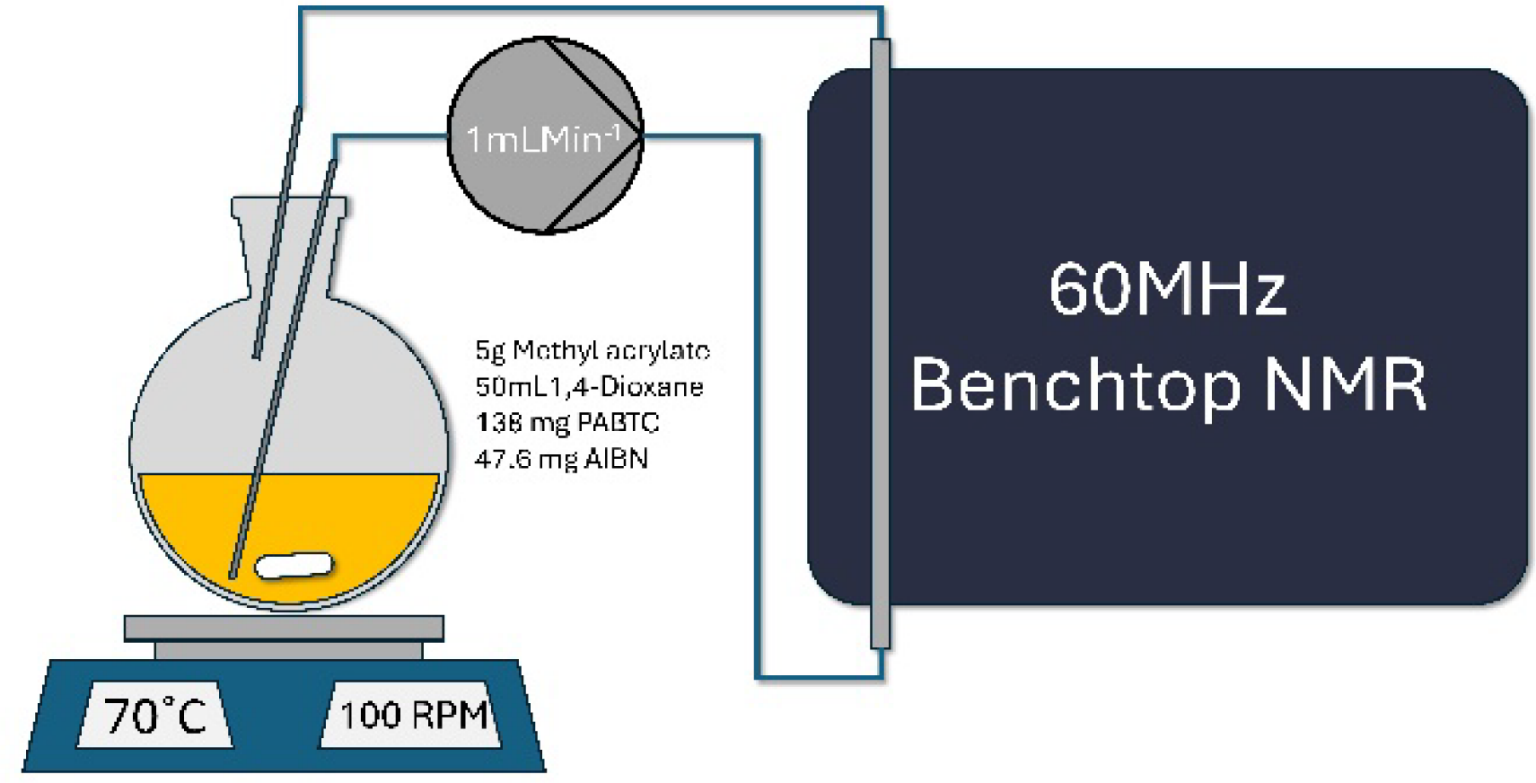
Schematic of the RAFT polymerization experimental set up.

The progress of the RAFT reaction was monitored as a function of time (Figure 4). As expected for a living polymerization, we observed an increase in molecular weight over time along with a corresponding increase in monomer conversion. The mass of the polymer continued to increase as the reaction progressed, reaching a maximum of ∼7000 Da at the termination of the reaction. Offline verification of the resulting mass of the polymer was conducted by GPC, yielding *M*_n_ = 4070 and *M*_w_ = 6470 g mol^–1^, based on a differential refractive index (DRI) detector and PMMA calibration with narrow molecular weight standards.

**Figure 4:**
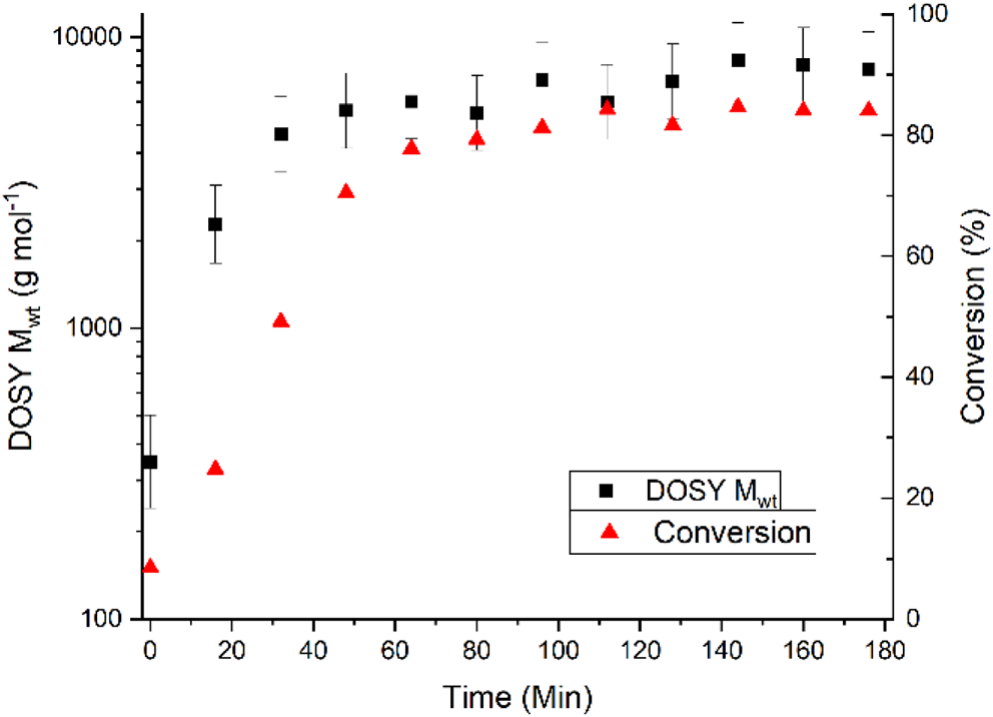
Polymerization of MA via thermally initiated RAFT as measured via continuous flow DOSY.

This reaction exhibits the expected kinetics of a typical living radical process, with a first-order rate constant of 1.27 ± 0.21 s^–1^, in agreement with literature values.^30^ Additionally, the 95% confidence intervals in these data are, throughout, not larger than 15%, similar to the typical uncertainties associated with GPC. This technique, however, is fully online and does not lose any sample during analysis.

We observe a good correlation between monomer conversion and expected product mass (Figure 5 ). The general trend of the experimentally determined masses matches the expected masses associated with a DP = 100 poly­(methyl acrylate) made by RAFT polymerization. As the polymerization reaches completion, there is a degree of variability within the determined mass; however, this theoretical mass is never outside the 95% confidence interval of any other measured value. This rate of monitoring is consistent with traditional online GPC; however, the conditions of these tests are only a start point for further optimization; therefore, it is not unrealistic to assume that the rate of data acquisition could be reduced, in line with stop-flow techniques described previously^17,18^

**Figure 5:**
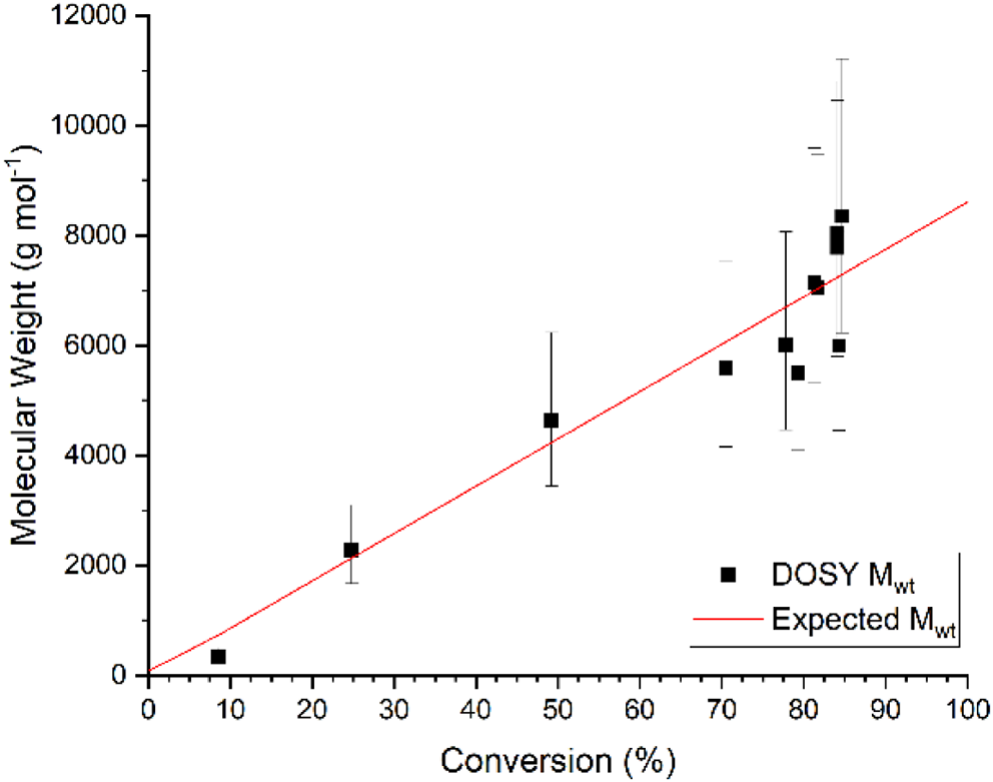
Molecular weight versus conversion for the RAFT polymerization of methyl acrylate.

Here, we have demonstrated a method to conduct MaDDOSY analysis under a range of continuous flow rates from 0.1 to 4 mL min^–1^, showing no correlation between the flow rate of the analyte and the resulting diffusion coefficient or *M*_D_. We also illustrate the application of this approach in monitoring an example of RAFT polymerization.

In this communication, we employed a limited range of polymers, molecular weights, and polymerization techniques, and we believe that this technique retains the universality of the original MaDDOSY calibration. Further work should aim to demonstrate MaDDOSY in continuous flow processes for a broader range of reaction mechanisms. This noninvasive and nondestructive technique shows promise for measuring polymer masses in continuous flow, with relevance to living polymerizations that yield narrow dispersity polymers, especially those that require moisture- and oxygen-free conditions. High polydispersity samples remain challenging to analyze accurately with this technique, and work is ongoing to not only provide more useful insight into these polymers but also, more generally, toward the facile determination of polydispersity.

This novel technique, which we have named mass acquisition by diffusion-in-flow ordered spectroscopy (MADFLOSY), and additional studies are planned to explore the full capabilities and limits of this approach for measuring molecular weight in the coming months and to demonstrate the broader utility of MADFLOSY for reaction monitoring in continuous flow chemistry.

## Supplementary Material



## Notes

Experimental procedures and spectra, materials and instrumentation (PDF)
