# Supplementary material for: MaDDFLOSY(Mass Determination via Diffusion in FLow Ordered SpectroscopY) for the Determination of Diffusion-Averaged Molecular Weight of Polymers in Continuous Motion Using Benchtop NMR
Source: Macromolecules. 2025 Apr 16;58(10):5201–7. doi: 10.1021/acs.macromol.4c03260 (PMC12120973; doi:10.1021/acs.macromol.4c03260)
Supplement: Supplementary file 1 [file ma4c03260_si_001.pdf]

Supporting Information

MaDDFLOSY(Mass Determination via diffusion in FLow Ordered SpectroscopY) for the determination of diffusion averaged molecular weight of polymers in continuous motion using benchtop NMR

William Pointer[a], Owen Tooley[a], Asad Saib[b], Rowan Radmall[a], Paul Wilson[a], Daniel Lester[a], James Town[a], Robin J. Blagg[b], and David Haddleton[a*]

a- Department of Chemistry, University of Warwick, Coventry CV4 7AL, United Kingdom; Polymer Characterization RTP, University of Warwick, Coventry CV47AL, United Kingdom

b- Oxford Instruments, Halifax Road, High Wycombe, Buckinghamshire, HP12 3SE, United Kingdom

Materials and Instrumentation

Styrene, methyl acrylate, methyl methacrylate, azobisisobutyronitrile (AIBN), 1,4-dioxane, poly(ethylene glycol) 3550 BioUltra, chloroform, tetrahydrofuran-d8, deuterium oxide, chloroform-d, tetrahydrofuran (THF), 2,6-Di-*tert*-butyl-4-methylphenol (BHT) and methanol were purchased from Merck. Poly(methyl methacrylate), and polystyrene standards (Agilent EasyVials) were purchased from Agilent.

The RAFT agent, 2-(((butylthio)carbonothioyl)thio)propanoic acid (PABTC), was synthesised in-house, according to previously reported literature.[1]

Benchtop NMR spectra were acquired on an Oxford Instruments X-Pulse Benchtop NMR spectrometer capable of producing a maximum gradient strength of ~570 mT m^{-1}. The sample temperature was held at 40°C. For 1D ^1H measurements a standard 90° pulse sequence was used, with 8 scans per experiment. For DOSY measurements, a J-compensated pulsed gradient stimulated echo pulse sequence was used for all acquisitions, with a little delta (δ) of 3 ms and big delta (Δ) of 300 ms. The number of scans for each step was 8, with 10 steps per experiment. Both 1D and DOSY benchtop spectra were interpreted using MestReNova Version 15.0.1-35756.

High field NMR spectra were acquired on a Bruker Avance 500 MHz spectrometer. The sample temperature was held at 40°C. 1D ^1H spectra were acquired using a standard zg30 pulse sequence with 16 scans. DOSY spectra were acquired using a ledbpgp2s pulse sequence with d20(Δ)= 100 ms and and p30(δ/2)=1.2 ms. The number of DOSY steps was 16, with 16 scans per step. Full acquisition parameters can be seen in figure 1. 1D ^1H spectra were interpreted using MestReNova Version 15.0.1-35756. DOSY spectra were interpreted using Bruker Topspin version 4.1.4.

GPC samples were run on an Agilent Infinity II MDS instrument equipped with differential refractive index (DRI), viscometry (VS), dual angle light scatter (LS) and multiple wavelength UV detectors. The system was equipped with 2 x PLgel Mixed C columns (300 x 7.5 mm) and a PLgel 5 µm guard column. The eluent was THF with 0.01% BHT additive. Samples were run at 1 ml/min at 30 °C. Poly(methyl methacrylate), and polystyrene standards (Agilent EasyVials) were used for calibration. Ethanol was added as a flow rate marker. Analyte samples were filtered through a GVHP membrane with 0.22 µm pore size before injection. Respectively, experimental molar mass (Mn, SEC) and dispersity (Đ) values of synthesized polymers were determined by conventional calibration using Agilent GPC/SEC software.

```
F2 - Acquisition Parameters
Date_          20241016
Time              15.31
INSTRUM           spect
PROBHD   5 mm BBO BB-1H
PULPROG       ledbpgp2s
TD                32768
SOLVENT             D2O
NS                   16
DS                    2
SWH           10416.667 Hz
FIDRES         0.317891 Hz
AQ            1.5728641 sec
RG                  512
DW               48.000 usec
DE                 6.50 usec
TE                313.0 K
D1           1.00000000 sec
D16          0.00020000 sec
D20          0.10000000 sec
D21          0.00500000 sec

======== CHANNEL f1 ========
NUC1                 1H
P1                13.25 usec
P2                26.50 usec
PL1                   0 dB
PL1W        17.28680229 W
SFO1        500.1330885 MHz

====== GRADIENT CHANNEL =====
GPNAM[6]       SINE.100
GPNAM[7]       SINE.100
GPNAM[8]       SINE.100
GPZ6             100.00 %
GPZ7             -17.13 %
GPZ8             -13.17 %
P19              600.00 usec
P30             1200.00 usec

F1 - Acquisition parameters
TD                   16
SFO1           500.1331 MHz
FIDRES       125.000000 Hz
SW                1.999 ppm
FnMODE               QF

F2 - Processing parameters
SI                32768
SF          500.1300000 MHz
WDW                  EM
SSB                   0
LB                -0.20 Hz
GB                    0
PC                 1.00

F1 - Processing parameters
SI                  128
MC2                  QF
SF          500.1300000 MHz
WDW                  no
SSB                   0
LB                    0 Hz
GB                    0
```

Figure 1 - Full high-field DOSY acquisition parameters

Polystyrene Synthesis

A 100 mL flask was charged with styrene (5 g, 48 mmol) dissolved in 1,4-dioxane (50 mL) and heated to 70 °C under constant stirring. Once at temperature, azobisisobutyronitrile (80 mg, 0.49 mmol) was added and the reaction allowed to proceed overnight. The resultant solution was added to ice-cold methanol to precipitate the product. The solvent was removed by vacuum filtration, and the precipitate allowed to dry for 48 hours to afford the title product as a white powder. GPC (THF): M_n = 10000 g mol^{-1}, M_w = 16700 g mol^{-1}, $Đ_M$ = 1.69 MaDDOSY: M_D (500 MHz, THF-d8) = 12500 g mol^{-1}, M_D (60 MHz, 1,4-dioxane) = 7600 g mol^{-1}.

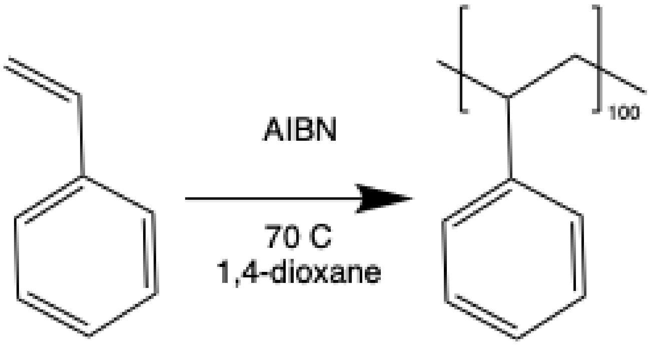

Scheme 1 - Free radical synthesis of polystyrene

Poly(methyl methacrylate) Synthesis

A 100 mL flask was charged with methyl methacrylate (5 g, 50 mmol) dissolved in 1,4-dioxane (50 mL) and heated to 70 °C under constant stirring. Once at temperature, azobisisobutyronitrile (80 mg, 0.49 mmol) was added and the reaction allowed to proceed overnight. The resultant solution was added to ice-cold methanol to precipitate the product. The solvent was removed by vacuum filtration, and the precipitate allowed to dry for 48 hours to afford the title product as a white powder. GPC (THF): M_n = 17800 g mol^{-1}, M_w = 45400 g mol^{-1}, $Đ_M$ = 2.55 MaDDOSY: M_D (500 MHz, $CDCl_3$) = 21900 g mol^{-1}, M_D (60 MHz, $CHCl_3$) = 37000 g mol^{-1}.

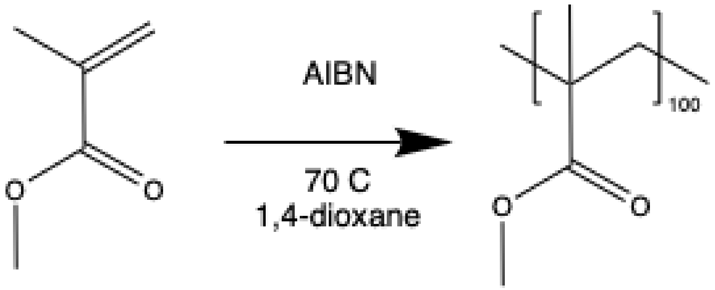

Scheme 2 - Free radical synthesis of PMMA

Polyethylene Glycol

Commercial poly(ethylene glycol) was used as is, but for completeness characterisation data is provided. GPC (THF): M_n = 5150 g mol^{-1}, M_w = 5490 g mol^{-1}, $Đ_M$ = 1.07 MaDDOSY: M_D (500 MHz, D_2O) = 5500 g mol^{-1}, M_D (60 MHz, H_2O) = 5400 g mol^{-1}.

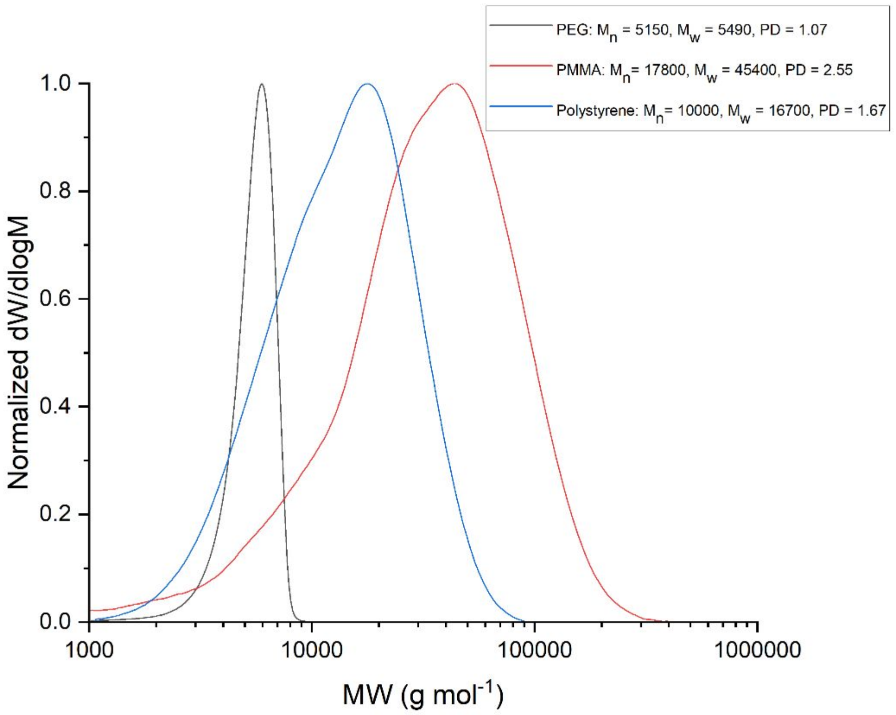

Figure 2 - GPC data for flow rate test polymers

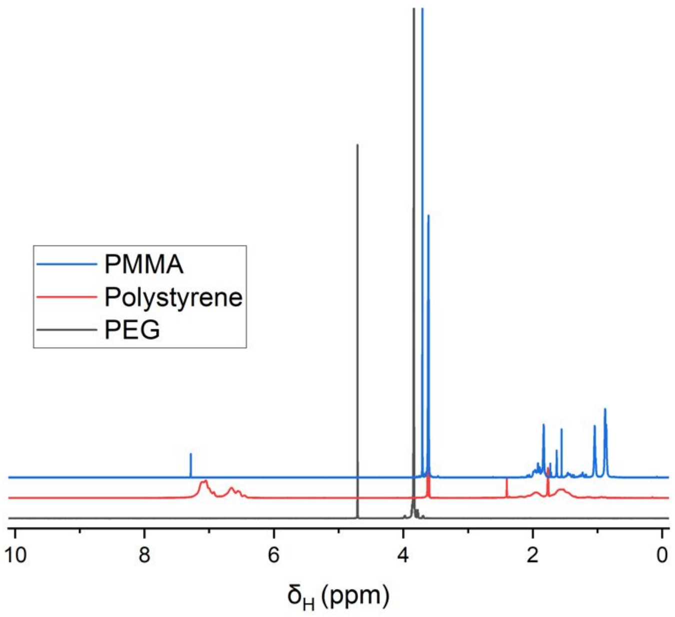

Figure 3 - 1D ^{1}H NMR data (500 MHz) of flow rate test polymers

Flow Rate Tests

Samples of the previously described poly(methyl methacrylate) (PMMA), polystyrene (PS) and poly(ethylene glycol) (PEG) were dissolved in chloroform, dioxane and water respectively at a concentration of 50 mg mL, these samples were then flowed through the Oxford Instruments X-Pulse spectrometer, equipped with a 4 mm glass flow cell using a master-flex peristaltic pump at 0.1, 0.25, 0.5, 1, 2 and 4 mL min^{-1}. Additionally, a static

measurement was performed in the flow cell to provide a spectrum with no flow applied to be used as a control.

Monitoring of the Synthesis of Poly(methyl acrylate) via a Thermally Initiated RAFT Mechanism using Flow-DOSY NMR

A 100 mL flask was charged with methyl acrylate (5 g, 58 mmol) dissolved in 1,4-dioxane (50 mL) this flask was degassed under nitrogen and heated to 70 °C using a thermostatted heating mantle. Once up to temperature, mixture was continuously flowed through the Oxford Instruments X-Pulse spectrometer, equipped with a 4 mm glass flow cell using a master-flex peristaltic pump at 1 mL min^{-1} both 1D and DOSY t0 measurements were taken. 2-(((butylthio)carbonothioyl)thio)propanoic acid (138 mg, 0.58 mmol) and azobisisobutyronitrile (47.6mg, 0.29 mmol) were added to the hot solution. The reaction was allowed to proceed for 180 minutes at 70 °C, under continuous flow through the magnet, during which 1D and DOSY measurements were made every 16 minutes. After this, the reaction was cooled in a freezer set to -20 °C and the solvent and residual monomer removed using evaporation with compressed air to afford the product as a viscous yellow oil. GPC (THF): M_n = 4070 g mol^{-1}, M_w = 6470 g mol^{-1}, $Đ_M$ = 1.58 MaDDOSY: M_D (500 MHz, THF-d8) = 8300 g mol^{-1}, M_D (60 MHz, 1,4-dioxane) = 7790 g mol^{-1}.

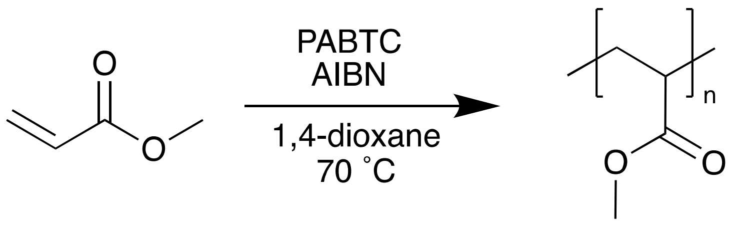

Scheme 3 - RAFT polymerization of methyl acrylate

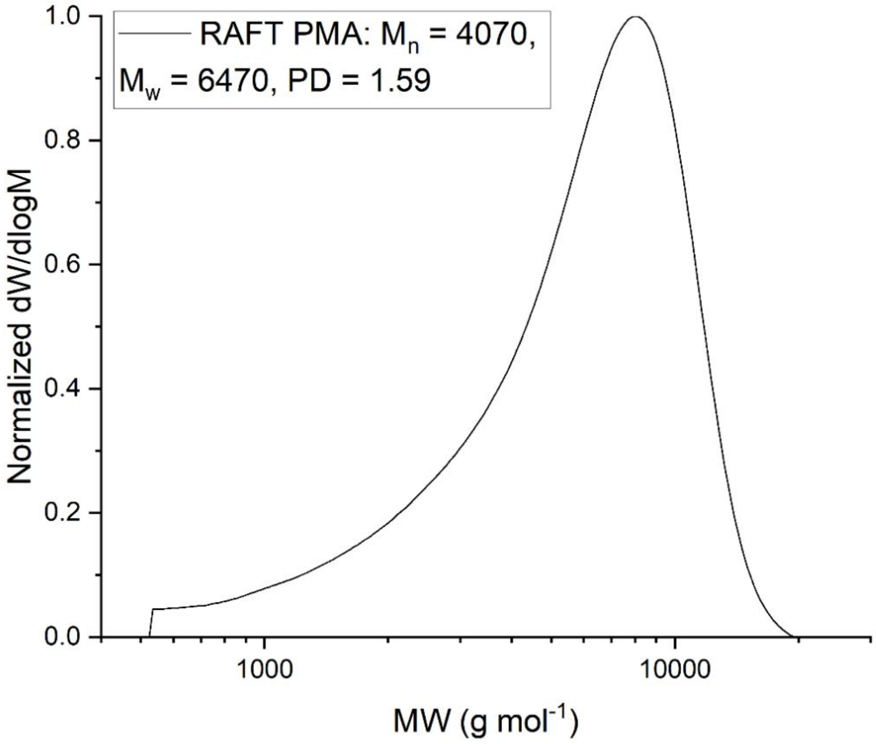

Figure 4 - RAFT poly(methyl acrylate) GPC chromatogram.

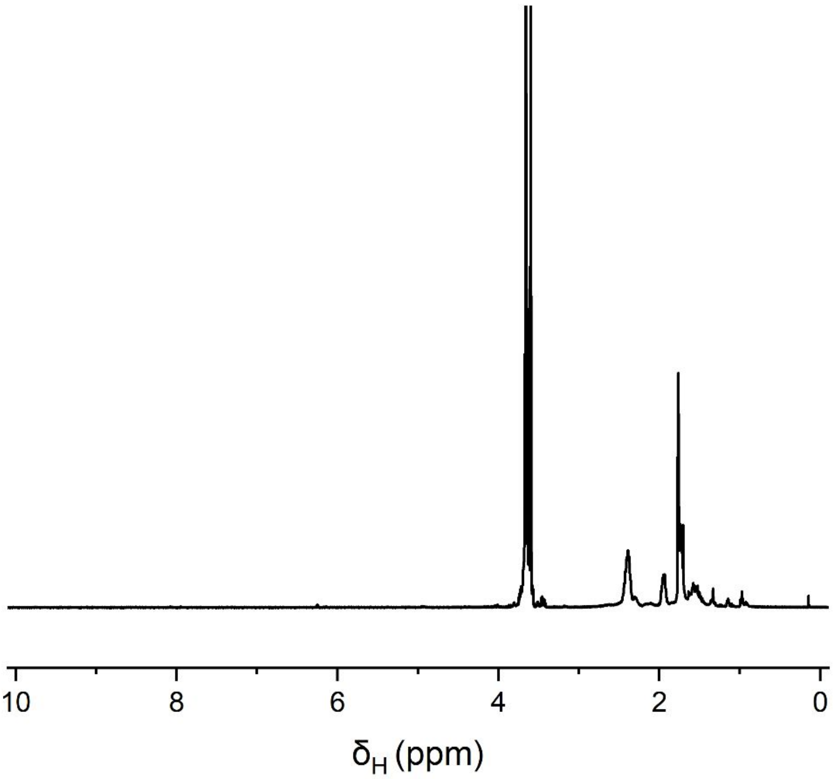

Figure 5 - 1D ^{1}H NMR spectrum of RAFT poly(methyl acrylate)

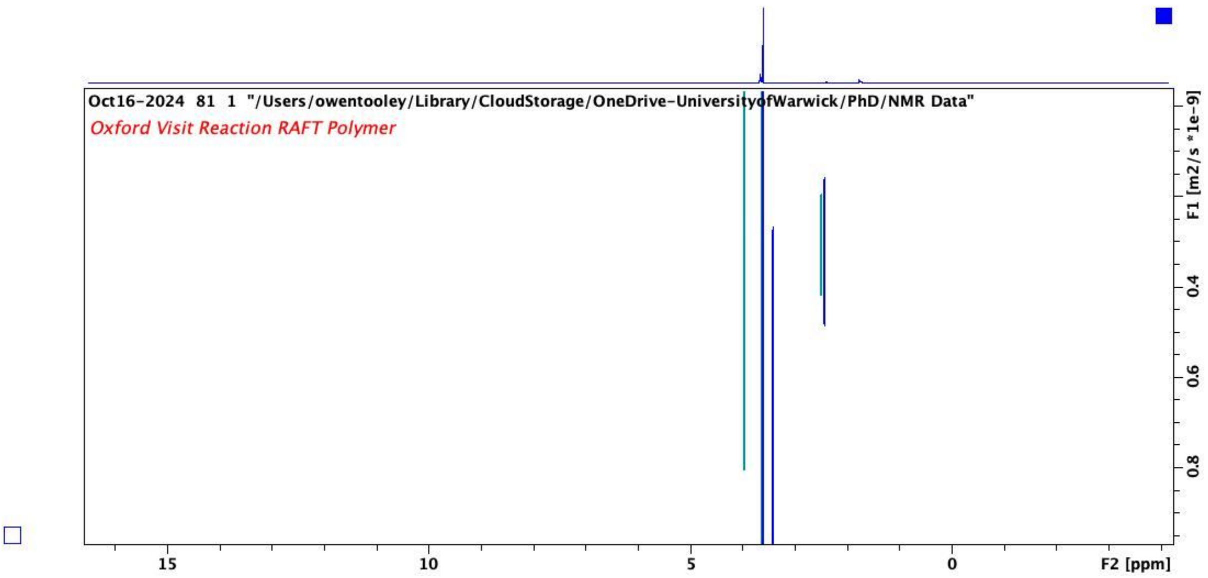

Figure 6 - 500 MHz DOSY of RAFT poly(methyl acrylate)

DOSY Analysis

In order to extract diffusion constants from the spectra acquired on the Oxford Instruments X-Pulse spectrometer, a Stejskal-Tanner plot must be made, showing peak integral as a function of relative gradient strength, as shown in figure 7.

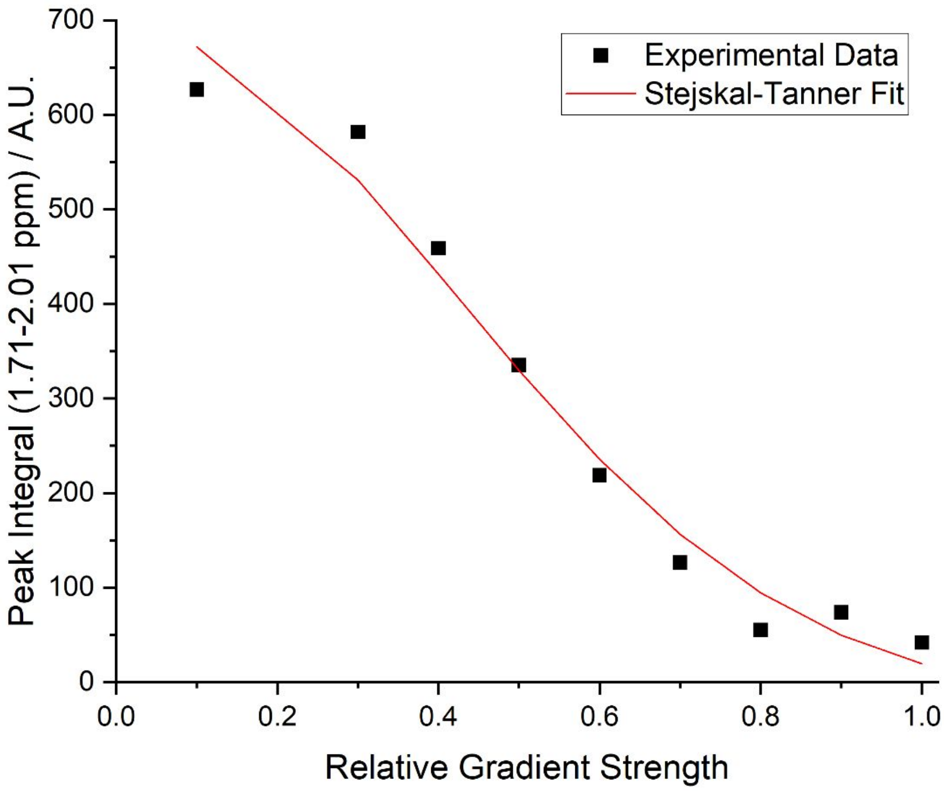

Figure 7 - Stejskal-Tanner plot for RAFT polymer t=16 min.

The Stejskal-Tanner plot can then be fit to equation 1, and the resultant value of S can be used to calculate the diffusion constant, as described in equation 2.[2]

$$y = Ie^{-x*x*S} + c$$

Equation 1 - Stejskal-Tanner fit equation

$$S = 2 \times \gamma^2 \times \delta^2 \times (Gmax)^2 \times (\Delta/2 - \delta/3) \times D$$

Equation 2 - Calculation of diffusion constant (D) from Stejskal-Tanner fit.

For completeness, representative spectra and associated Stejskal-Tanner fits are shown in figures 8-10. In each case, some points have been removed from the fit if there were phasing issues around the analyte peak that could not be corrected in post-processing. These were particularly common in the 0-gradient step. These could be addressed in future work by accumulating more scans at each step or running at higher concentrations, which were closer to C* as determined in our previous work.

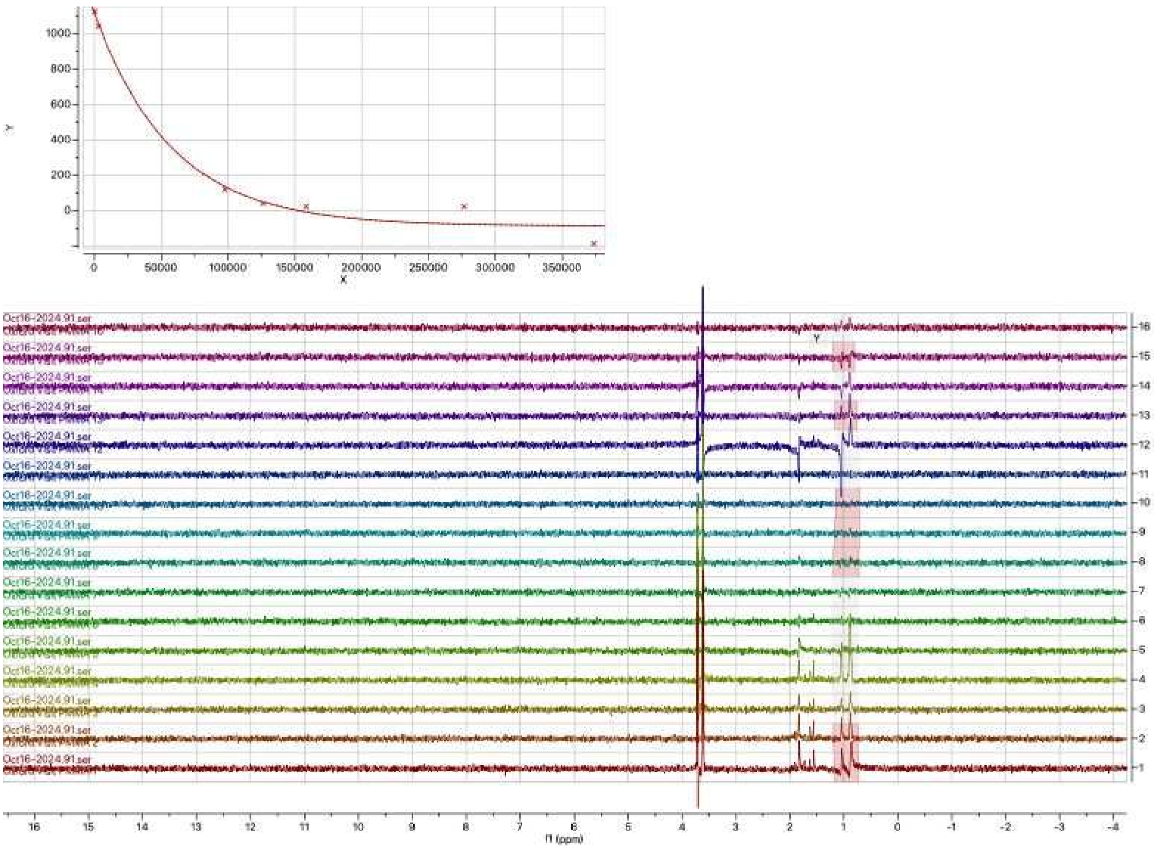

Figure 8 - DOSY spectra and Stejskal-Tanner plot for PMMA.

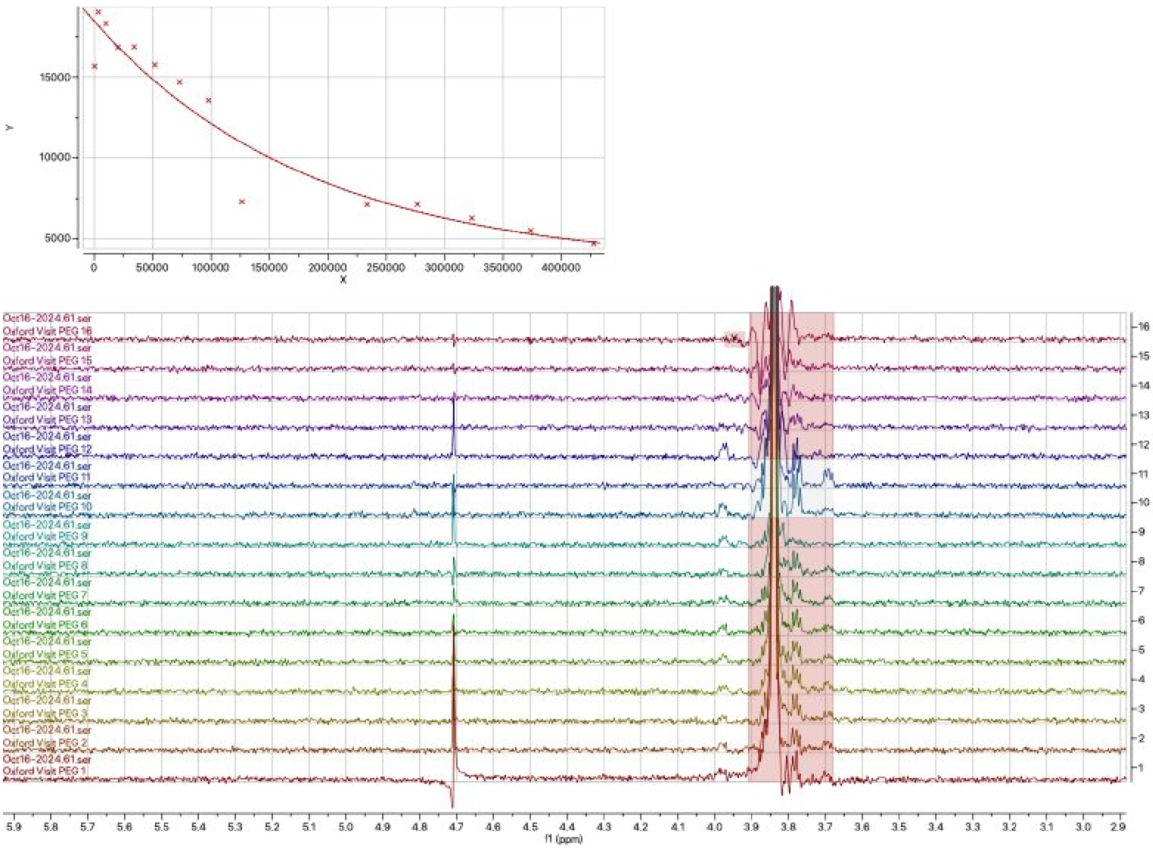

Figure 9 - DOSY spectra and Stejskal-Tanner plot for PEG.

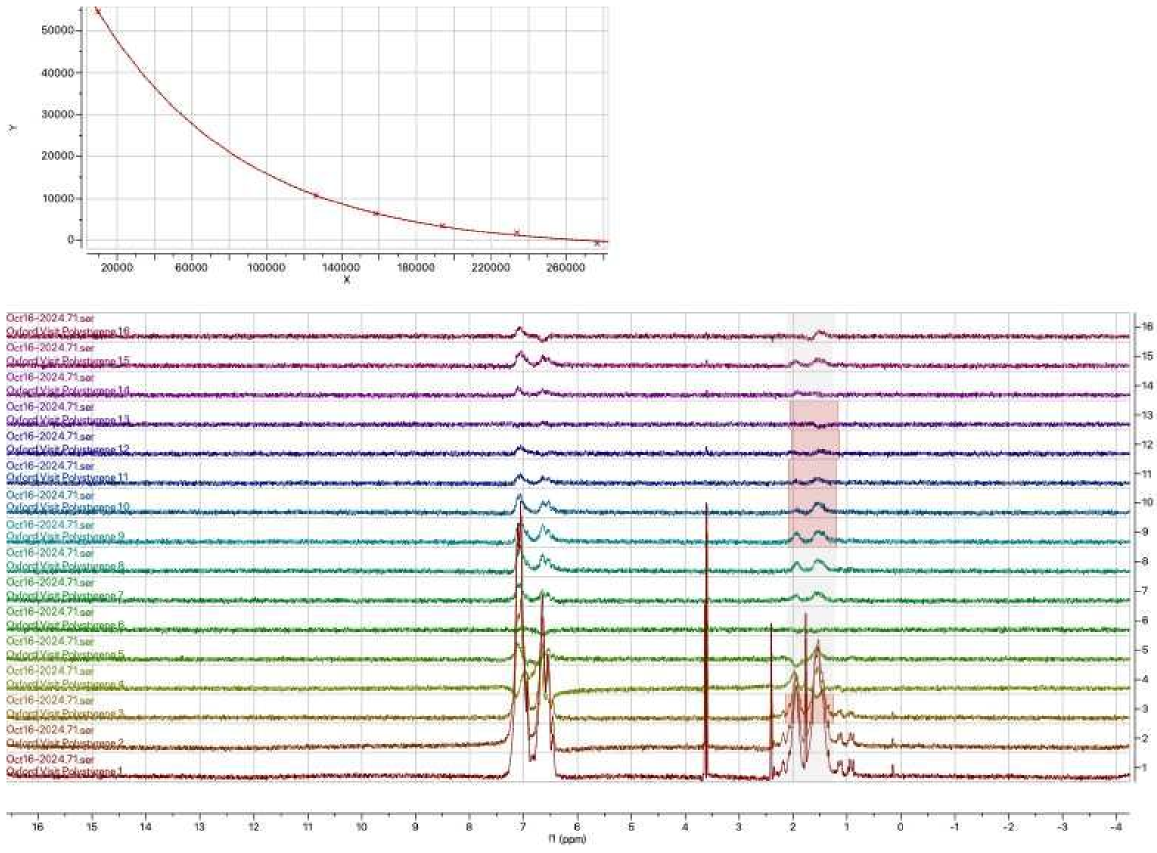

Figure 10 - DOSY spectra and Stejskal-Tanner plot for polystyrene.

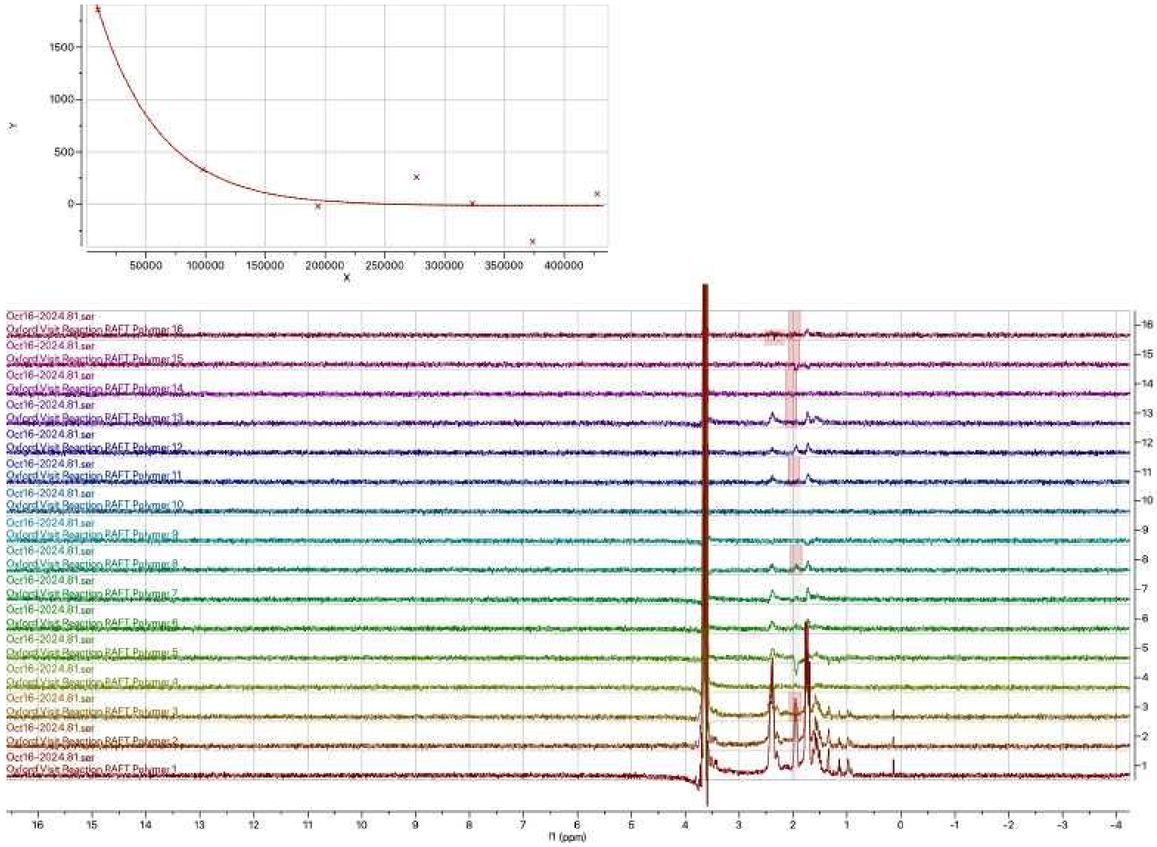

Figure 11 - DOSY spectra and Stejskal-Tanner plot for RAFT poly(methyl acrylate).

For the DOSY spectra acquired on the Bruker Avance 500 MHz spectrometer, fitting was performed using topspin version 4.1.4. A Contin processing model was used, to fit either 1 or 2 parameters, dependent on the intensity of the solvent peak. A maximum of 250 iterations were performed with the calculated diffusion constant constrained between $1x10^{-12}$ and $1x10^{-9}$ m^2 s^{-1}, the expected values for a free polymer in solution at 40°C. For completeness, representative spectra acquired in this way are shown for PEG (figure 12) and the RAFT poly(methyl acrylate) (figure 6).

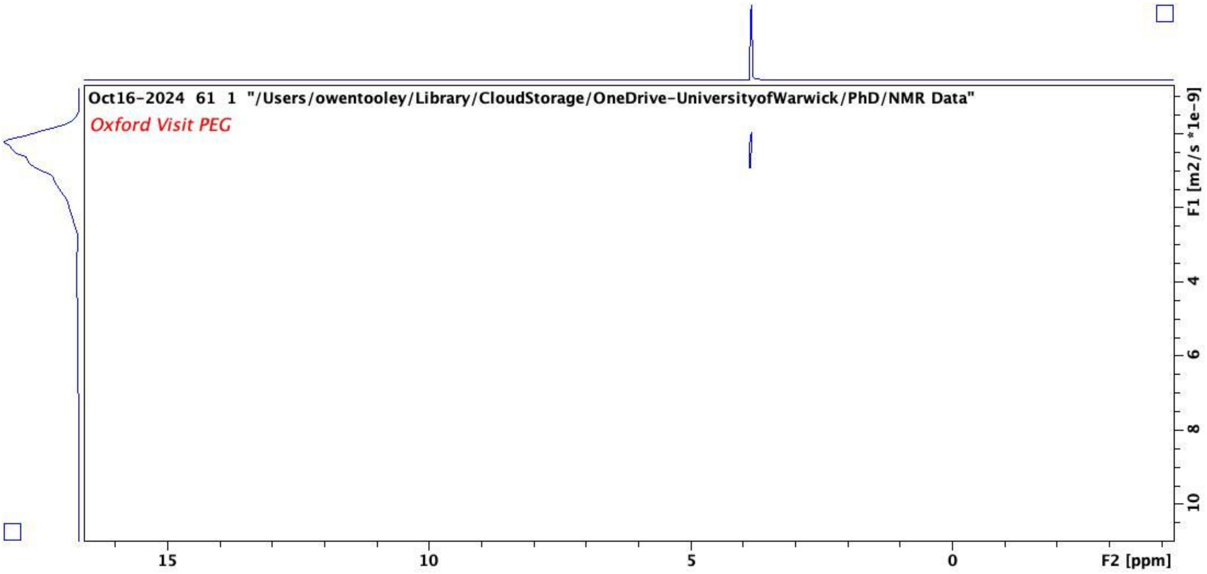

Figure 12 - 500 MHz 2D DOSY projection for PEG.

Temperature Correction

Poly(ethylene glycol) (15.1 mg) was dissolved in deuterium oxide (755 uL) and transferred to an NMR tube. DOSY spectra were acquired as previously described using a Bruker Avance 500 spectrometer, equipped with a variable temperature probe. Spectra were acquired at both 26.5 ˚ C and 40 ˚ C. The diffusion constant acquired at 26.5 ˚ C was $2.25x10^{-10}$ m^2 s^{-1} and at 40 ˚ C was $4.1725x10^{-10}$ m^2 s^{-1}. These gave hydrodynamic radii of 0.7795 and 0.7872 nm respectively. By then back calculating the effective diffusion constant for the 40 ˚ C sample if it were at 26.5 ˚C using the Stokes-Einstein equation, and relevant solvent viscosity of D_2O, the result is $2.2325x10^{-10}$ m^2 s^{-1}, which is in very close agreement with the experimentally measured value, allowing us to be confident that this temperature correction is appropriate for small changes in temperature.

Supporting Information References

(1) Ferguson, C. J.; Hughes, R. J.; Nguyen, D.; Pham, B. T. T.; Gilbert, R. G.; Serelis, A. K.; Such, C. H.; Hawkett, B. S. Ab Initio Emulsion Polymerization by RAFT-Controlled Self-Assembly. Macromolecules 2005, 38 (6), 2191–2204. https://doi.org/10.1021/ma048787r.

(2) Torres, A. M.; Zheng, G.; Price, W. S. J-Compensated PGSE: An Improved NMR Diffusion Experiment with Fewer Phase Distortions. Magn. Reson. Chem. 2010, 48 (2), 129–133. https://doi.org/10.1002/mrc.2555.
